# Whole blood miRNA expression signatures as predictors of neoadjuvant chemotherapy response in triple-negative breast cancer

**DOI:** 10.1186/s41065-026-00712-8

**Published:** 2026-07-08

**Authors:** Monika Drobniene, Domas Stitilis, Linas Kunigenas, Egle Strainiene, Sonata Jarmalaite, Kestutis Suziedelis

**Affiliations:** 1 Laboratory of Molecular Oncology, National Cancer Institute, Vilnius, Lithuania; 2 Institute of Biosciences, Life Sciences Center, Vilnius University, Vilnius, Lithuania; 3 Department of Chemistry and Bioengineering, Vilnius Gediminas Technical University, Vilnius, Lithuania; 4 Clinical Research Center, National Cancer Institute, Vilnius, Lithuania

**Keywords:** TNBC, miRNAs, Non-invasive clinical biomarkers, Neoadjuvant chemotherapy, Survival

## Abstract

**Background:**

Triple negative breast cancer (TNBC) is an aggressive malignancy distinguished by resistance to standard breast cancer therapies. The absence of reliable prognostic tools hinders the ability to predict therapeutic response, resulting in some patients receiving treatment that is ineffective yet physically exhausting. miRNAs are small non-coding RNA molecules that regulate gene expression, and their dysregulated profiles in liquid biopsies hold immense potential as minimally invasive biomarkers for monitoring treatment response.

**Results:**

In this study, we seek to identify miRNAs that may serve as predictive biomarkers for therapeutic responses in patients diagnosed with triple-negative breast cancer. To achieve this, an in silico analysis of the Cancer Genome Atlas (TCGA) dataset is conducted to narrow the list of candidate miRNAs. miRNAs with potential involvement in TNBC were selected for clinical validation in whole-blood samples of TNBC patients before and after therapy. The RT-qPCR-based expression analysis revealed significant predictive potential of *miR-340-5p*, *miR-1307-3p*, *miR-185-5p*, and *miR-30a-3p*.

**Conclusions:**

Our study identified that dysregulated miRNA signatures can serve as independent indicators for therapeutic monitoring. Notably, the combined multi-miRNA expression panel offers a robust, superior predictive value for stratifying treatment response and tracking disease progression in TNBC patients.

**Supplementary Information:**

The online version contains supplementary material available at https://doi.org/10.1186/s41065-026-00712-8.

## Introduction

 Known for its aggressive nature, triple-negative breast cancer (TNBC) accounts for 10–15% of all breast cancer cases [1, 2]. This type of cancer is associated with a higher relapse risk than other breast pathologies [3]. Because TNBC lacks expression of estrogen (ER) and progesterone (PR) receptors and does not exhibit HER2 overexpression or amplification, endocrine therapy and HER2-directed therapies are ineffective in the neoadjuvant, adjuvant, or metastatic setting. Instead, chemotherapy and immunotherapy are administered preoperatively and may be continued postoperatively. Tumor response to therapy is evaluated after surgery, and the treatment plan is adjusted accordingly. In case of cancer resistance, alternative strategies are used. However, if there were markers of cancer resistance that could be identified in advance, a number of patients would not have to go through exhausting therapies that yield no improvements.

miRNAs are small, typically ~ 22 nucleotide length non-coding RNAs responsible for gene expression regulation by primarily binding to 3’ untranslated regions and blocking translation or promoting degradation. By targeting genes involved in the cell cycle, apoptosis, and DNA repair, miRNAs can significantly influence carcinogenesis, resulting in either suppression or inhibition of tumor development. Research on breast cancer shows serum miRNA deregulation compared to healthy controls and a correlation between tissue and serum miRNA expression [4]. Importantly, breast cancer subtypes exhibit distinct patterns of miRNA expression, which may serve as markers for these subtypes, including TNBC [5]. Therefore, whole blood-derived miRNAs are a promising approach for biomarker research, providing a more comprehensive representation of systemic miRNA expression [6, 7]. This approach simplifies the sample collection process and minimises variation in preparation compared with serum, which is affected by blood clotting or different handling procedures [7, 8]. Nevertheless, while numerous candidate miRNAs have been associated with treatment response and resistance [9, 10], none have yet transitioned to routine, clinically standardised use. This study aims to identify candidate miRNAs with potential clinical applicability to predict treatment outcomes and personalise treatment strategies in TNBC.

## Methods

### Selection of miRNA for expression analysis

In order to select a number of candidate miRNAs, potentially involved in therapy response for triple negative breast cancer (TNBC), genomic data from The Cancer Genome Atlas (TCGA) were analysed [11]. miRNA microarray and gene sequencing data were retrieved using cBioPortal [12–14]. Data from 526 gene sequencing samples were retrieved, of which 89 were from patients negative for estrogen, progesterone, and HER2 markers. We also used data of 297 breast cancer patient tissue microarrays, of which 48 were of TNBC. Relative expression analysis was conducted using tools provided by cBioPortal. Samples were divided into two groups: triple-negative (estrogen receptor negative, progesterone receptor negative, and HER2 negative) and non-triple-negative. Statistical significance of relative gene and miRNA expression was assessed using Student’s t-test and Benjamini – Hochberg correction. miRNAs or genes were considered significantly differentially expressed if the q-values were less than 0,05. When a specific miRNA arm was not indicated in the miRNA microarray data, both possible mature arms were considered for further analysis.

**Fig. 1:**
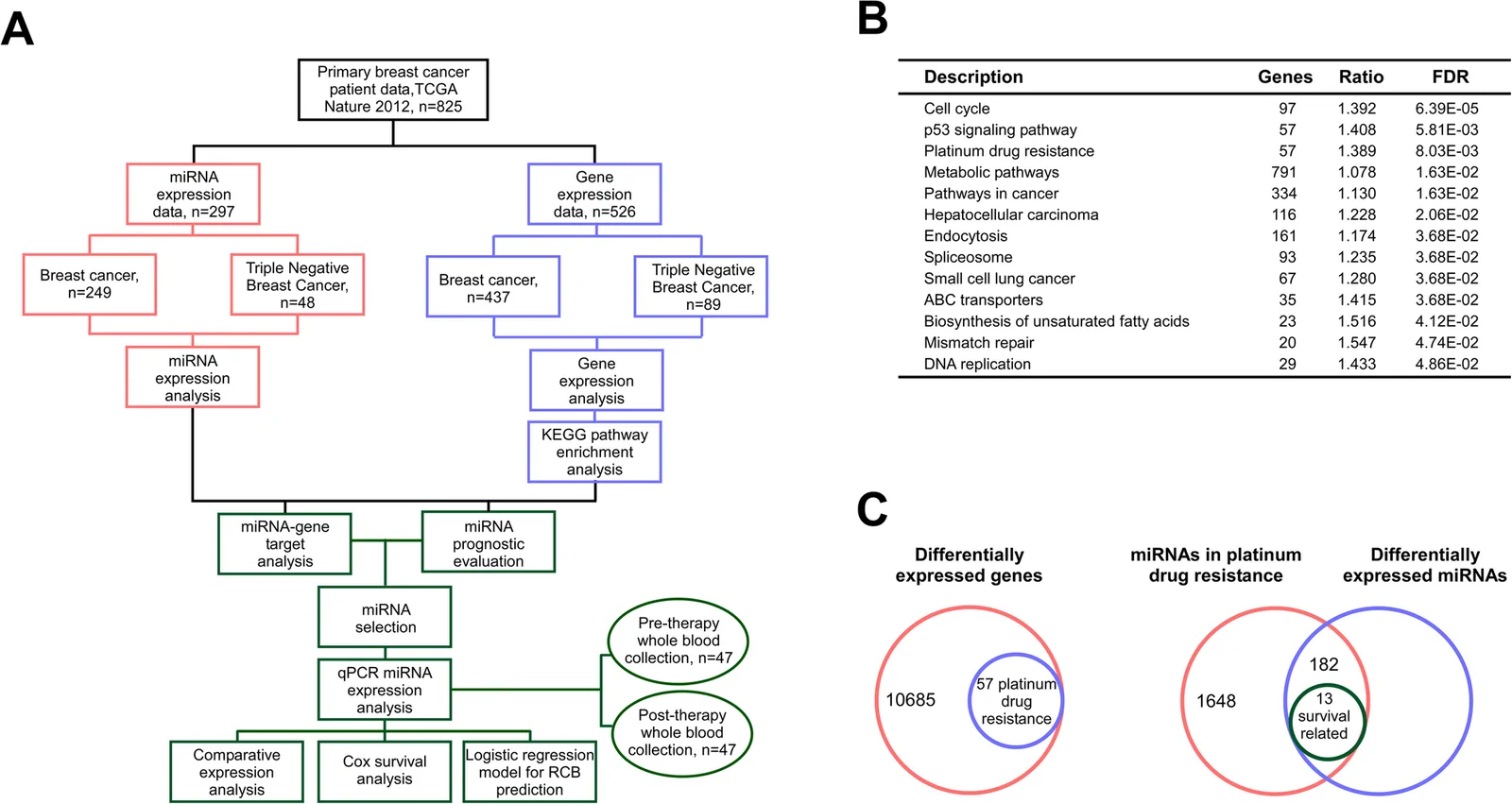
**A** diagram outlining processes for candidate miRNA selection and expression analysis in whole blood TNBC patient samples; **B** KEGG pathway enrichment analysis of deregulated genes in TNBC samples; **C** Venn diagrams depicting miRNA selection by integrating gene expression, miRNA expression, KEGG pathway and patient survival data

Differentially expressed genes were submitted to KEGG pathway enrichment analysis using the WebGestalt online tool [15]. miRNAs targeting specific genes involved in the platinum drug resistance pathway were identified using the DIANA-TarBase v8 database [16]. The relationship between miRNA expression levels and patient survival was analysed using the OncoLNC online tool [17]. Log-rank tests were performed to compare survival between patients in the top third of miRNA expression and those in the bottom third. A protein-protein and gene-miRNA interaction network was constructed using the Cytoscape application StringApp [18]. A graphical abstract of the data processing pipeline, outlining major steps, is shown in Fig. 1A.

### Study population and samples

This single-centre study included 47 eligible patients aged 18–75 years with histologically confirmed stage II–III triple-negative breast cancer (TNBC) diagnosed between 2019 and 2022. TNBC was defined by the absence of estrogen receptor and progesterone receptor expression and the lack of HER2 overexpression or amplification. All patients had an Eastern Cooperative Oncology Group (ECOG) performance status < 2. Patients were excluded if they had received any previous systemic or local treatment for breast cancer, had severe uncontrolled comorbid conditions, or had clinically significant organ dysfunction that could preclude the administration of systemic therapy. Besides, they could not participate in any other interventional clinical trial.

All patients provided written informed consent to participate in the study. All clinical procedures were carried out at the National Cancer Institute (NCI) in Lithuania in accordance with the Helsinki Declaration. The study received approval from the Institutional Scientific Review Board of the NCI (protocol code II-2018-1) and the Vilnius Regional Bioethics Committee (Approval number 2019/2-1084-589). All patients received standard neoadjuvant chemotherapy, undergoing 12 weekly cycles of paclitaxel (80 mg/m^2^) and carboplatin (AUC 1.5-2), followed by four 3-weekly cycles of AC (doxorubicin 60 mg/m^2^ plus cyclophosphamide 600 mg/m^2^). Two whole-blood samples from 47 patients (*n* = 94) were collected into Paxgene tubes (Qiagen) before neoadjuvant chemotherapy and after chemotherapeutic treatment, 3–5 weeks before surgical tumor resection, and stored at − 70 °C until needed. Surgery was performed in all patients, and radiotherapy was administered in accordance with local guidelines. Adjuvant treatment with capecitabine was administered to 9 of 47 patients with residual disease. The patient’s clinical and pathologic characteristics are summarised in Table 1. The follow-up period was 49 months, from enrollment to the end of the study.

**Table 1:** Clinicopathologic characteristics of the patients

	Responders	Poor-responders
Variables		
Patient number	25	22
Patient age at diagnosis, years		
Average	54.72	52.82
Median	57	53.5
Range	32–72	32–70
Menopausal status, patient number (percent)		
Premenopausal	10 (40%)	10 (45.5%)
Postmenopausal	15 (60%)	12 (54.5%)
Clinical stage, patient number (percent)		
II	17 (68%)	11 (50%)
III	8 (32%)	11 (50%)
Tumor category (cT), patient number (percent)		
T1	6 (24%)	4 (18.18%)
T2	16 (64%)	14 (63.64%)
T3	2 (8%)	0
T4	1 (4%)	4 (18.18%)
Node positivity (cN), patient number (percent)		
0	10 (40%)	4 (18.2%)
1	9 (36%)	9 (40.9%)
2	5 (20%)	6 (27.3%)
3	1 (4%)	3 (13.6%)
Histologic grade (G), patient number (percent)		
G1	2 (8%)	0
G2	0	6 (27.3%)
G3	23 (92%)	16 (72.7%)
Proliferation index Ki67 (%), patient number (percent)		
< 60%	11 (%)	8 (36.4%)
≥ 60%	14 (%)	14 (63.6%)
Median % (range)	60% (3–95%)	60% (4–95%)
Treatment, patient number (percent)		
Neoadjuvant chemotherapy	25 (100%)	22 (100%)
Surgery	25 (100%)	22 (100%)
Adjuvant chemotherapy (capecitabine)	0	9 (40.9%)
Adjuvant radiotherapy	20 (80%)	19 (86.4%)
Pathological response, patient number (percent)		
Complete response (CR)	14 (56%)	0
Partial response (PR)	11 (44%)	16 (72.72%)
Stable disease (SD)	0	3 (13.64%)
Locally progressive disease (PD)	0	3 (13.64%)
Residual cancer burden (RCB class), patient number (percent)		
0	14 (56%)	0
1	11 (44%)	0
2	0	12 (54.5%)
3	0	10 (45.5%)
Survival data, patient number (percent)		
Disease progression	2 (8%)	12 (54.5%)
Deceased	2 (8%)	8 (36.4%)
Follow-up, months	0–48	0–49
Median overall survival (OS), months	42	25
Median progression-free survival (PFS), months	42	22

### Pathological assessment

Pathological diagnosis based on standard immunohistochemical (IHC) staining was performed in an accredited pathology laboratory using clinically validated assays and quality control procedures on primary tumor tissue samples collected by a core needle biopsy to determine the status of hormonal receptors (ER, PR, HER2), and the Ki67 proliferation index was assessed. The following primary antibodies were used: estrogen receptor (ER), rabbit monoclonal SP1 (Ventana); progesterone receptor (PR), rabbit monoclonal 1E2 (Ventana); and HER2, rabbit monoclonal 4B5 (Ventana). All tumors were considered to have negative hormonal receptor status if they had less than 1% stained cells for ER and PR. HER2 receptor status was evaluated as negative with an IHC score of 0/1+; for HER2 equivocal cases (scored 2+), fluorescence in situ hybridisation (FISH) analysis was conducted, revealing no HER2 gene amplification. HER2 FISH analysis and interpretation were performed according to ASCO/CAP recommendations. According to the median score, Ki67 expression was rated as high if ≥ 60% of cells were positive on IHC. Breast pathologists evaluated the response to neoadjuvant chemotherapy postoperatively. Pathological complete response (pCR) was defined as the absence of invasive cancer in the breast and lymph nodes (ypT0/in situ, ypN0). An incomplete response after neoadjuvant chemotherapy (non-pCR) refers to the presence of residual cancer cells in breast tissue and/or lymph nodes at the time of surgery. A residual cancer burden (RCB) score was calculated using a validated methodology and the Residual Cancer Burden Calculator [19]. This score was then used to define the RCB class for further prognostic evaluation. The patients were separated into two groups according to response to treatment and further assessment of prognostic factors (RCB0 (pCR), RCB-1, and RCB-2/3). Patients with pathological complete response (pCR) and RCB-1 were considered to have a favorable response to NACT (responders), whereas those with RCB-2/3 were considered to have a poor response (poor-responders) [20, 21].

### miRNA isolation from the patient’s whole blood sample

Total miRNA was extracted from 47 TNBC patients of pre- and post-therapy whole-blood samples using PAXgene Blood miRNA Kit (PreAnalytiX, Qiagen). All whole-blood samples were stored uniformly at -70 °C for at least 2 months, and RNA was extracted from each patient’s pre- and post-therapy samples concurrently in a single extraction run. Total isolated miRNA was further quantified using a NanoDrop spectrophotometer (Thermo Fisher Scientific, USA). The quantity of isolated miRNA in each sample was within a range of 2.75–13 µg.

### miRNA expression analysis

Further, miRNA relative expression in the patient’s whole blood sample was evaluated using RT-qPCR. cDNA was prepared using the RevertAid RT Reverse Transcription Kit (Thermo Fisher Scientific) according to the manufacturer‘s instructions. A total of 0.2 µg of miRNA from each sample was converted to cDNA and kept in − 20 °C till further analysis. Briefly, each reaction mixture included 4 µL of reaction buffer, 0.8 µL of dNTP mix, 0.8 µL of 0.25 µM stem-loop RT primer, 0.4 µL of Ribolock RNase inhibitor, 200 ng of RNA, 12 µL of nuclease-free water, and 200 U of RevertAid Reverse Transcriptase. The expression level of candidate microRNA was evaluated using Luminaris HiGreen qPCR Master Mix (ThermoFisher Scientific) according to the manufacturer’s instructions, and RT-qPCR was performed using QuantStudio™ 5 Real–Time PCR System (ThermoFisher Scientific). Briefly, for each reaction in a 96-well plate, 1 µL of 4-fold-diluted cDNA, 5 µL Luminaris HiGreen qPCR Master Mix, 3.4 µL nuclease-free water, and 0.3 µM each of the forward and reverse universal primers were used. The cycling condition for RT-qPCR reaction was initial activation at 95 °C for 3 min, following cycling conditions (3 cycles): denaturation (95 °C for 15 s), annealing (55 °C for 60 s), and extension (60 °C for 30 s) and then followed by 40 cycles of 2-step amplification consisting of denaturation (95 °C for 10 s), annealing, and extension ( 60 °C 30s). Primer sequences used for miRNA expression analysis are shown (Supplementary files 1, Table “primer sequences”). The method for real-time quantification of miRNA according to [22]. The expression levels of miRNA were analysed in relation to the expression of the endogenous control RNU48 [6, 23] ( Supplementary file 2, Table “melting and amplification curves”). To minimise the chances of error, each sample was assessed in triplicate, and the 2^−ΔCt^ method was used to calculate the results [24].

### Statistical analysis

Data analysis and visualisation were performed using GraphPad Prism software (v8.0; GraphPad Software, Inc.). The normality of the data distribution was verified using the Shapiro–Wilk W-test. To evaluate differences in miRNA expression levels between paired pre- and post-therapy whole-blood samples, a paired *t-*test was used. For clinical sub-analyses, patients were stratified into two groups based on clinicopathological evaluation: a residual cancer burden of 0–1 (RCB 0–1) and a residual cancer burden of 2–3 (RCB 2–3). For all analyses, differences were considered statistically significant when the *p*-value was less than 0.05 (*p* < 0.05).

### Multiple comparisons of miRNA expression and clinicopathological characteristics

The ANOVA test was employed for multiple comparisons between miRNA expression levels and clinicopathological characteristics. Normality of data was assessed using the Shapiro – Wilk normality test. To evaluate data variance, the Levene test for homogeneity of variance was employed. ANOVA tests were employed to assess differences in miRNA expression across various clinicopathological characteristics for data that passed normality and homogeneity of variance tests. For clinicopathological characteristics with more than 2 levels, Tukey’s tests were done. Welch’s one-way test was employed for data with nonhomogeneous variance. For non-normal data, the Kruskal–Wallis rank-sum test was performed, and for characteristics with more than 2 levels, pairwise comparisons using the Wilcoxon rank-sum exact test were employed. All procedures were performed using R 4.3.0 software.

### Survival analysis

Cox proportional hazards regression analysis was performed using the survival_3.7-0 package in R. Both univariate and multivariate analyses were executed. Time to death or time to progression were used as the outcome variables. For multivariate analysis, patients’ age, cancer stage, Ki-67% proliferation index, tumour grade, and RCB group were selected as clinicopathological factors. Reproduction status was excluded from the analysis due to a direct association with patients’ age. Cancer stage was retained as a factor rather than cT and cN, which are used to derive it. Lasso penalized Cox regression model was used for variable selection with 10-fold cross-validation. To account for the small clinical cohort size, 1000 bootstrap iterations were performed to assess variable robustness. Variables for which the coefficients were not penalised to zero coefficients in at least 70 per cent of iterations were further used to build Cox models. Resulted models were tested for multicollinearity using the variance inflation factor test and for hazard ratio stability over time using the Schoenfeld residual test. Clinicopathological factors were considered statistically significant if the *p*–value was less than 0.05.

### RCB prediction model construction using logistic regression

A logistic regression model with an LASSO penalty was applied to identify miRNAs that discriminate between RCB0-1 and RCB2-3 groups. Model tuning and variable selection were performed using 10-fold cross-validation. The selected miRNAs were then used to build generalised regression models from which the apparent area under the curve (AUC) was estimated. To assess model optimism, we used a bootstrap resampling approach with 1000 iterations. In each iteration, bootstrap samples were randomly selected for model training, and model performance was evaluated on the full dataset to compute AUC. Optimism penalties were calculated by subtracting the AUC obtained in each bootstrap iteration from the apparent AUC. Final optimism penalty was defined as the mean value of the resulting optimism penalty distribution. All analyses were performed using R_4.3.0 and the packages pROC (version 1.18.5) and glmnet (version 4.1-8).

## Results

### Identification of dysregulated miRNA in triple negative breast tumors

Relative gene expression analysis of The Cancer Genome Project data revealed 10,742 differentially expressed genes (Fig. 1, Supplementary file 3). Further pathway enrichment analysis revealed 13 significantly enriched KEGG pathways, including Cell cycle, p53 signaling pathway and platinum drug resistance, the latter was further inspected (Fig. 1.B). miRNA target analysis revealed 1843 miRNAs that target differentially expressed platinum drug resistance gene group. Of those miRNAs, 195 were deregulated in triple negative breast cancer patient samples. Although subsequent Cox regression analysis did not show a correlation between miRNA expression levels and patient survival time, the log-rank test revealed 13 miRNAs associated with significantly different survival times between patients with the lowest and highest miRNA expression levels (Supplementary file 3, Tab. “platinum_BC_survival”).

### miRNA expression in triple negative breast cancer patient samples

**Fig. 2:**
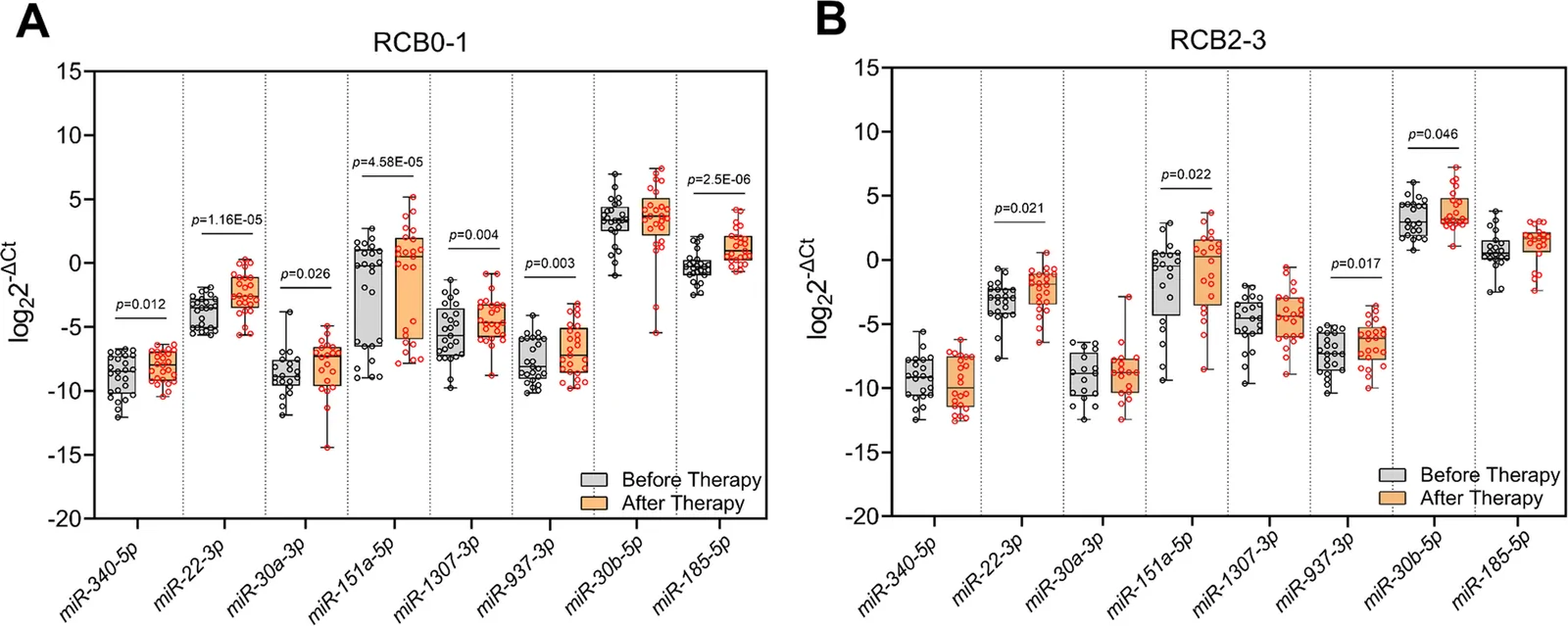
Relative expression of *miR-340-5p*, miR-22-3p, *miR-30a-3p*, *miR-151a-5p*, *miR-1307-3p*, *miR-937-3p*, *miR-30b-5p*, *miR-185-5p* in TNBC group RCB 0–1 (**A**) (*n* = 25) and RCB2-3 (**B**) (*n* = 22) whole blood samples compared before and after NAC therapy, detected by RT-qPCR. Expression levels of miRNAs were determined by the 2^−ΔCt^ method. Data normality was verified using the Shapiro–Wilk *W*-test, followed by a paired *t*-test to evaluate the statistical significance of differences between the groups. Whiskers of the boxplot denote the non-outlier range

A panel of miRNAs - including *miR-340-5p*, *miR-22-3p*, *miR-30a-3p*, *miR-151a-5p*, *miR-1307-3p*, *miR-937-3p*, *miR-30b-5p*, *miR-185-5p* - was analyzed in the whole blood of TNBC patients before and after neoadjuvant chemotherapy (NAC) using RT-qPCR (Fig. 2). These miRNAs were selected to further investigate whether they could provide valuable insights into chemoresistance to conventional anthracycline/taxane based chemotherapy including platinum drugs, cancer progression and poor survival of TNBC patients.

The expression levels of *miR-22-3p* (*p* = 1.16E-05) and *miR-151a-5p* (*p* = 4.58E-05) increased significantly after therapy (Fig. 2A) in the RCB0-1 patient group, suggesting a dynamic regulation in response to treatment. The notable increase in *miR-22-3p* expression within the RCB0-1 group indicates a potential association with a favorable therapeutic response. In contrast, in patients with poor treatment responses (RCB2-3) exhibited only a minor, yet statistically significant, increase in the expression of *miR-340-5p* and *miR-151a-5p* (Fig. 2B). This indicates the presence of resistance-associated profiles. In contrast, *miR-340-5p* (*p* = 1.2E-02) and *miR-30a-3p* (*p* = 2.57E-02) showed a significant upregulation only in the RCB0-1 group, suggesting a tandem action by deactivating platinum resistance and reversing EMT processes [25, 26]. In particular, higher *miR-340-5p* expression has been shown to suppress tumor growth and is linked to more favorable clinical outcomes [27]. In contrast, *miR-340-5p* and *miR-30a-3p* showed no significant change in expression between pre- and post-treatment samples in patients with high residual cancer burden (RCB 2–3) (Fig. 2B), suggesting limited involvement of these miRNAs in modulating chemotherapy response within this patient subgroup.

The expression of *miR-185-5p* indicates a “rebound” or increase in whole blood levels after the chemotherapeutic treatment (Fig. 2A). This increase correlates with their identification as potential predictors of treatment outcome and was observed only in the RCB 0-1- group, which is opposite to RCB2-3, as there was no difference in *miR-185-5p* expression after the NAC.

The expression patterns of *miR-1307-3p*, *miR-937-3p*, and *miR-30b*-5p indicate that changes after NAC are linked more to tumor presence and chemoresistance than to outcomes of treatment itself (Fig. 2A, B). These miRNAs’ expression may signal tumor biology and survival outcomes rather than therapy efficacy. This implies that the impact of chemotherapy on miRNAs reflects variations in cancer-related molecular mechanisms, rather than a direct response to the treatment. Overall, these findings highlight the potential significance of these miRNAs in the pathogenesis of triple-negative breast cancer (TNBC) and support the need for further investigation into their predictive potential in triple-negative breast cancer.

### Comparative miRNA expression analysis

Multiple testing analysis identified several miRNAs differentially expressed between residual cancer burden groups RCB0-1 and RCB2-3 (Supplementary file 4). Analysis of primary tumor extent (cT) revealed deregulation of *miR-151-5p* before therapy (*p* = 0.045). Stratification by lymph node status (cN) demonstrated significant differences in *miR-30a-3p* expression change between cN1 and cN2 (*p* = 0.04), as well as reduced *miR-30b-5p* expression in cN2 compared to cN0 before therapy (*p* = 0.012). Similarly, post-therapy levels of *miR-30b-5p* were lower in both cN1 (*p* = 0.03) and cN2 (*p* = 0.013) in contrast to cN0. Additionally, *miR-340-5p* expression prior to therapy was reduced in cN2 relative to cN0 (*p* = 0.016) and cN1 (*p* = 0.027). *miR-1307-3p* was associated with patient survival and disease progression, as patients who died or experienced disease progression within four years exhibited higher *miR-1307-3p* expression before therapy (death: *p* = 0.021; progression: *p* = 0.021). Ki67 levels were associated with the expression of *miR-185-5p* both before (*p* = 0.042) and after therapy (*p* = 0.014), as well as with post-treatment levels of *miR-22-3p* (*p* = 0.006).

### Prognostic value of miRNA expression in triple negative breast cancer: a cox regression study

In the univariate Cox regression analysis, the pre-therapy expression levels of *miR-1307-3p* and *miR-30b-5p*, as well as the post-therapy expression levels of *miR-937-3p* and *miR-185-5p*, were significantly associated with overall patient survival (Supplementary file 5). Clinicopathological factors significantly associated with survival included RCB2-3 and patient age.

However, multivariate Cox analysis of pre-therapy miRNA expression and clinicopathological factors revealed that only *miR-1307-3p* (HR = 2.3, 95% CI: 1.45–3.65) and *miR-340-5p* (HR = 0.53, 95% CI: 0.32–0.86) remained independently associated with patient survival, alongside RCB (HR = 14.52, 95% CI: 1.91–110.71) and age (HR: 0.93, 95CI: 0.88–0.99) (Supplementary file 6). Notably, when post-therapy miRNA expression levels or longitudinal changes in expression were introduced into multivariate models, no miRNAs retained significance; only RCB and patients’ age remained independent prognostic factors for survival. Several miRNAs were also significantly associated with time to cancer progression in the univariate analysis. These included pre-therapy expression of *miR-1307-3p* (HR = 1.549, 95% CI: 1.142–2.101) and *miR-30b-5p* (HR = 1.475, 95% CI: 1.046–2.078), as well as post-therapy expression of *miR-1307-3p* (HR = 1.394, 95% CI: 1.043–1.863), *miR-30b-5p* (HR = 1.371, 95% CI: 1.028–1.828), *miR-937-3p* (HR = 1.422, 95% CI: 1.036–1.952) and *miR-185-5p* (HR = 1.596, 95% CI: 1.041–2.448).

Clinicopathological factors univariately associated with shorter time to cancer progression included RCB2-3 (HR = 9.219, 95% CI: 2.053–41.39), cN3 lymph node status (HR = 7.312, 95% CI: 1.218–43.921) and cancer stage 3 disease (HR = 3.168, 95% CI: 1.061–9.464). In the multivariate Cox models for cancer progression, pre-therapy analysis demonstrated that both RCB (HR = 29.08, 95% CI: 4.341–194.809) and *miR-1307-3p* (HR = 2.087, 95% CI: 1.385–3.145) were independent predictors. In the post-therapy multivariate model, RCB remained a significant cofactor RCB (HR = 12.693, 95% CI: 2.059–64.221), alongside *miR-185-5p* (HR = 2.658, 95% CI: 1.352–5.224), *miR-1307-3p* (HR = 1.497, 95% CI: 1.013–2.21), Ki67 index (HR = 1.07, 95% CI: 1.029–1.112), and patient age (HR = 0.941, 95% CI: 0.887–0.999). Finally, in the model assessing dynamic changes in miRNA expression over time, no miRNA changes reached statistical significance, and only RCB (HR = 9.82, 95% CI: 2.168–44.484) and Ki67 index (HR = 1.023, 95% CI: 1–1.046) remained as independent predictors of progression.

### ROC analysis of single and 4 miRNA model for distinguishing patients into residual cancer burden groups

**Fig. 3:**
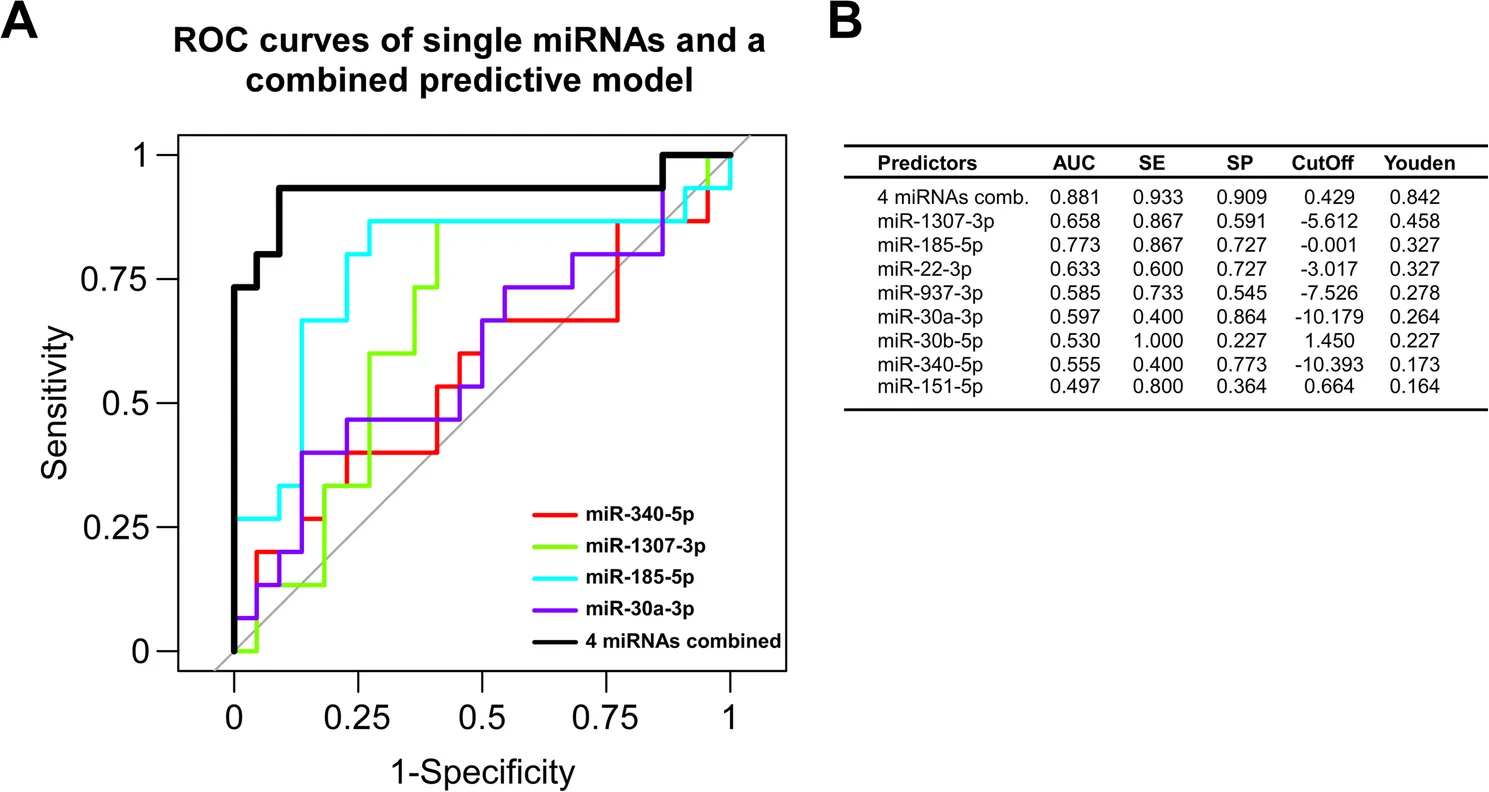
**A** figure depicting ROC curves of the top 4 miRNAs and a combined 4 miRNA signature before neoadjuvant therapy. Combined model consists of *miR-340-5p*, *miR-1307-3p*, *miR-185-5p*, and *miR-30a-3p*. Risk score = -15.0547 – (1.218 * *miR-340-5p*) + (0.411 * *miR-1307-3p*) + (1.791 * *miR-185-5p*) – (0.621 * *miR-30a-3p*); **B** ROC analysis result table of 8 miRNAs and a 4 miRNA signature

To evaluate the potential of miRNAs as predictive biomarkers of tumor response to treatment, individual miRNAs and a group of 4 of the best performing miRNAs were assessed for their ability to distinguish patients into categories by residual cancer burden. Pre-therapy miRNA expression levels were selected as a more valuable data time point for predicting the therapeutic effect. When analysed individually, miRNAs exhibited modest to no discriminatory capacity. Among them, *miR-185-5p* showed the strongest performance, achieving an area under the curve (AUC) of 0.773. However, a 4 miRNA combination (*miR-1307-3p, miR-185-5p, miR-30a-3p* and *miR-340-5p*) provided a better discriminatory ability between the RCB0-1 and RCB2-3 groups, achieving an apparent AUC of 0.927. After 1000 bootstrap iterations, the calculated model optimism penalty was 0.0464, which reduced the AUC to 0.881 (95% CI: 0.755–1) (Fig. 3A, Supplementary figure S1).

## Discussion

The present study aimed to investigate changes in circulating miRNA levels in the blood of triple negative breast cancer patients undergoing neoadjuvant therapy and their relationship with clinicopathological factors. We particularly anticipated differences in miRNA expression between TNBC patients with weak (RCB2–RCB3) or strong (RCB0–RCB1) responses to neoadjuvant therapy, in the hope of revealing the potential of miRNAs to predict patients’ responses to therapy. In the first step of candidate miRNA selection, we filtered miRNAs associated with drug resistance and TNBC patient survival. The platinum drug resistance pathway was prioritised for the miRNA filtering strategy, as identifying miRNAs associated with resistance to platinum compounds may help identify patients who might not benefit from treatment and may suffer from additional toxicity. We confirmed their importance in TNBC by demonstrating characteristic differences in expression levels across several clinicopathological factors. As expected, pre-therapy levels of *miR-1307-3p* expression were associated with increased risk of disease progression and death. In addition, these miRNA levels before therapy, with the addition of *miR-340-5p*, *miR-185-5p* and *miR-30a-3p*, showed superior ROC results for distinguishing patients with strong or weak responses to neoadjuvant therapy. However, as singular prognostic factors, miRNAs demonstrated limited predictive power; only *miR-185-5p* showed some distinction, albeit with modest effectiveness.

Triple negative breast cancer is notable for its resistance to conventional drug therapies. The backbone of neoadjuvant chemotherapy used in TNBC is a standard anthracycline/taxane regimen. For decades, there has been debate about the addition of platinum compounds due to controversial data. Based on the evidence, current guidelines suggest that adding carboplatin to neoadjuvant chemotherapy may be considered to increase the likelihood of achieving a pCR, balancing potential benefit against the higher risk of hematologic toxicity [28].

In this study, we demonstrate that specific miRNAs associated with drug resistance can also serve as markers for more resistant TNBC cases. Previous studies highlight selected miRNAs’ oncogenic or tumour suppressive roles in cancer biology. For instance, *miR-1307-3p* has been reported to promote breast cancer cell proliferation, tumor growth and angiogenesis [29, 30]. In contrast, *miR-185-5p* is typically down-regulated in breast cancer tissues, and its overexpression has been shown to enhance radiosensitivity, inhibit lung metastasis and induce apoptosis [31–33]. Our analysis of TCGA data reveals that *miR-185-5p* is significantly overexpressed in TNBC tissues and is associated with a higher risk of progression and mortality. Other studies suggest that *miR-185-5p* may facilitate the transformation of adjacent normal fibroblasts into cancer-associated fibroblasts via extracellular vesicle-mediated transport, potentially accounting for its higher levels in TNBC patients’ tissues and blood [34]. *miR-30a-3p* is shown to be down-regulated in TNBC tumor tissues, and its overexpression inhibits epithelial-mesenchymal transition, proliferation and cell migration [35]. While low *miR-30a-3p* expression level is associated with poor survival, our study did not find a significant relationship between its expression and progression or mortality [36]. Interestingly, *miR-30b-5p* is also down-regulated in breast cancer tissue, but circulating levels are elevated in the blood of cancer patients, particularly in those with invasive and metastatic disease [37]. *miR-937-3p* plays a role in breast cancer by promoting cell invasiveness and migration [38]. This coordinated dysregulation of *miR-1307-3p*, *miR-30b-5p*, *miR-937-3p*, and *miR-185-5p* in whole blood not only reduces intracellular platinum concentrations but also promotes the epithelial-to-mesenchymal transition (EMT), thereby equipping cells to withstand cytotoxic stress [10]. Contrary to this, *miR-340-5p* functions as a tumor suppressor by inhibiting proliferation and drug resistance through targeting LGR5 [39].

Several studies have evaluated the diagnostic potential of miRNAs in breast cancer detection. Analysis of miRNA levels in the serum of breast cancer patients has demonstrated the diagnostic utility of a panel that includes *miR-1307-3p* [40]. While building a miRNA panel for early breast cancer detection, a model comprising *miR-5100*, *miR-1307-3p* and *miR-4745-5p* was identified as the most effective, based on a substantial cohort of 11,349 patients [41]. Working with a significantly smaller dataset of early breast cancer patients’ blood resulted in a model of 240 miRNAs to diagnose disease [42]. The study of TNBC patients’ serum was also successful in identifying a prognostic miRNA panel predicting cancer recurrence, comprising the miRNAs *miR-18b*, *miR-103*, *miR-107*, and *miR-652* [43].

Although relatively stable miRNA expression does not necessarily rule out diagnostic relevance, we implemented specific criteria to identify candidate miRNAs with functional significance [42]. Specifically, we prioritised miRNAs that were differentially expressed in TNBC tissues compared with other breast cancer subtypes. To further refine our candidate miRNA pool, miRNAs that remained after initial selection were subjected to additional filtering based on the biological roles of their target genes and their association with patient survival outcomes. Although integrating transcriptional data and functional annotations may have improved the effectiveness of miRNA selection, this strategy may have excluded miRNAs with limited information on their roles in TNBC or those relevant to specific patient subgroups. Also, the limited sample size of TNBC patients in our study restricted more thorough testing, which may have impacted the findings. Therefore, future research involving larger cohorts is warranted to better assess the predictive value of miRNAs in TNBC.

## Conclusions

Currently, the response to treatment during neoadjuvant therapy is assessed clinically and radiologically, and in some cases, may not be fully informative. This approach limits the ability to appropriately select effective drugs or adjust during the course of therapy. The definitive assessment of breast cancer response is performed after the surgery. miRNAs are emerging as promising diagnostic and prognostic biomarkers in the clinical setting. Distinct patterns of miRNA expression have been shown to be specific to certain cancers and their subtypes, underscoring their potential utility in oncology. Circulating miRNAs are notably stable in body fluids and are secreted actively by cells or released as a result of cell death. Moreover, sample acquisition for circulating miRNA testing is a minimally invasive procedure. Therefore, monitoring miRNA expression levels in blood before therapy could be employed to predict tumor response to therapy, improving outcomes of TNBC patients.

## Supplementary Information

Supplementary Material 1.

Supplementary Material 2.

Supplementary Material 3.

Supplementary Material 4.

Supplementary Material 5.

Supplementary Material 6.

Supplementary Material 7.

## Data Availability

All data supporting the findings of this study are available within the paper and its Supplementary Information.
