# Supplementary material for: Whole blood miRNA expression signatures as predictors of neoadjuvant chemotherapy response in triple-negative breast cancer
Source: Hereditas. 2026 Jul 8;163:102. doi: 10.1186/s41065-026-00712-8 (PMC13632267; doi:10.1186/s41065-026-00712-8)
Supplement: Supplementary file 1 [file 41065_2026_712_MOESM1_ESM.docx]

**Supplementary 1**

**Table 1.** miRNA accession numbers, primers and designed RT stem-loops.

| **miRNA** | **Accession number,**  miRbase (https://www.mirbase.org/) | **Specific forward primer** | **RT stem-loop primer** |
| --- | --- | --- | --- |
| *miR-340–5p* | MIMAT0004692 | GCGGCGTTATAAAGCAATGAGA | GTCGTATCCAGTGCAGGGTCCGAGGTATTCGCACTGGATACGACAATCAG |
| *miR-1307–3p* | MIMAT0005951 | ACTCGGCGTGGCGTCG | GTCGTATCCAGTGCAGGGTCCGAGGTATTCGCACTGGATACGACCACGAC |
| *miR-937–3p* | MIMAT0004980 | GATCCGCGCTCTGACTC | GTCGTATCCAGTGCAGGGTCCGAGGTATTCGCACTGGATACGACGGCAGA |
| *miR-30a–3p* | MIMAT0000088 | GCGCTTTCAGTCGGATGTT | GTCGTATCCAGTGCAGGGTCCGAGGTATTCGCACTGGATACGACGCTGCT |
| *miR-30b–5p* | MIMAT0004589 | GGCTGTAAACATCCTACAC | GTCGTATCCAGTGCAGGGTCCGAGGTATTCGCACTGGATACGACAGCTGA |
| *miR-185–5p* | MIMAT0000455 | GCTGGAGAGAAAGGCAGT | GTCGTATCCAGTGCAGGGTCCGAGGTATTCGCACTGGATACGACTCAGGA |
| *miR-22–3p* | MIMAT0000077 | GCGAAGCTGCCAGTTGAAG | GTCGTATCCAGTGCAGGGTCCGAGGTATTCGCACTGGATACGACACAGTT |
| *miR-151a–5p* | MIMAT0004697 | GCTCGAGGAGCTCACAG | GTCGTATCCAGTGCAGGGTCCGAGGTATTCGCACTGGATACGACACTAGA |
| RNU48 (SNORD48) | NR_002745.1** | GCGAGCACAGAATTAATACGAC | TGATGATGACCCCAGGTAACTCT |

** GenBank (<https://www.ncbi.nlm.nih.gov>)

Universal Reverse Primer: CAGTGCAGGGTCCGAGGT (The generated cDNA is amplified by PCR with a miRNA-specific forward primer and a universal reverse primer).
