# Supplementary material for: Whole blood miRNA expression signatures as predictors of neoadjuvant chemotherapy response in triple-negative breast cancer
Source: Hereditas. 2026 Jul 8;163:102. doi: 10.1186/s41065-026-00712-8 (PMC13632267; doi:10.1186/s41065-026-00712-8)
Supplement: Supplementary file 2 [file 41065_2026_712_MOESM2_ESM.docx]

| *miR-340-5p* | *miR-340-5p* |
| --- | --- |
| *miR-22-3p* | *miR-22-3p*  |
| *miR-1307-3p*  | *miR-1307-3p*  |
| *miR-937-3p* | *miR-937-3p*  |
| *miR-30a-3p* | *miR-30a-3p*  |
| *miR-30b-5p* | *miR-30b-5p*  |
| *miR-185-5p*  | *miR-185-5p*  |
| *miR-151a-5p* | *miR-151a-5p*  |
| *RNU48* | *RNU48*   |
