# Supplementary material for: Whole blood miRNA expression signatures as predictors of neoadjuvant chemotherapy response in triple-negative breast cancer
Source: Hereditas. 2026 Jul 8;163:102. doi: 10.1186/s41065-026-00712-8 (PMC13632267; doi:10.1186/s41065-026-00712-8)
Supplement: Supplementary file 3 [file 41065_2026_712_MOESM7_ESM.docx]

**Supplementary figure S1.** A nomogram of 4 miRNA panel (miR-1307-3p, miR-185-5p, miR-30a-3p and miR-340-5p) predicting TNBC patients residual cancer burden group.
